# CausalFormer-HMC: a hybrid memory-driven transformer with causal reasoning and counterfactual explainability for leukemia diagnosis

**DOI:** 10.3389/fcell.2025.1674393

**Published:** 2025-10-13

**Authors:** Fares Jammal, Mohamed Dahab

**Affiliations:** Department of Computer Science, Faculty of Computing and Information Technology, King Abdulaziz University, Jeddah, Saudi Arabia

**Keywords:** acute lymphoblastic leukemia (ALL), causal-former-HMC, hybrid deep learning, peripheral blood smear classification, explainable AI in medical imaging

## Abstract

Acute Lymphoblastic Leukemia (ALL) is a prevalent malignancy particularly among children. It poses diagnostic challenges due to its morphological similarities with normal cells and the limitations of conventional methods like bone marrow biopsies, which are invasive and resource-intensive. This study introduces Causal-Former-HMC, a novel hybrid AI architecture integrating convolutional neural networks, vision transformers, and a causal graph learner with counterfactual reasoning to enhance diagnostic precision and interpretability from peripheral blood smear (PBS) images. We utilized two robust datasets: the ALL Image collection, comprising 89 patients and 3,256 PBS images (504 benign, 2,752 malignant across Pro B, Pre B, and Early Pre B subtypes), and C-NMC dataset, containing 15,135 segmented cell images from 118 patients (7,272 leukemic, 3,389 normal). To address class imbalance, we implemented class-aware data augmentation, standardizing image counts across classes and resizing to 128 × 128 pixels for compatibility with our model. The proposed model is evaluated via stratified 5-fold cross-validation with Nadam, SGD, and Radam (fractional) optimizers, Causal-Former-HMC achieved perfect classification accuracy (100%) and macro-averaged F1-scores on the ALL dataset, and up to 98.5% accuracy with 0.9975 ROC-AUC on the C-NMC dataset hence demonstrating superior generalization. Interpretability was ensured through advanced explainable AI techniques, including Grad-CAM, LIME, Integrated Gradients, and SHAP, which consistently highlighted attention to clinically relevant features such as nuclear contour irregularities and chromatin condensation. These results underscore the potential of the model to deliver non-invasive, accurate and transparent diagnostics that pave the way for its integration into clinical hematology workflows and advancing AI-driven leukemia screening paradigms. Index Terms—Acute Lymphoblastic Leukemia (ALL); Causal-Former-HMC; Hybrid Deep Learning; Peripheral Blood Smear Classification; Explainable AI in Medical Imaging.

## 1 Introduction

Acute lymphoblastic leukemia (ALL), a prevalent condition, particularly in children, accounts for around 25% of paediatric malignancies (Ward et al., 2014). Because malignant and normal cells have structural similarities, diagnosing it can be difficult and frequently necessitates invasive procedures like bone marrow biopsies, that can be resource-intensive and mostly rely on expert interpretation (Bhojwani et al., 2015). These traditional methods may cause delays in diagnosis and treatment, which can affect patient outcomes, particularly in settings with limited resources. A possible avenue for creating non-invasive, precise, and interpretable diagnostic tools to address these problems is the advent and development of deep learning (DL), a subset of artificial intelligence (AI) (Wu and Gadsden, 2022).

In order to improve diagnosis accuracy from peripheral blood smear (PBS) images, this study introduces Causal-Former-HMC, an innovative hybrid AI module that integrates vision transformers (ViTs), convolutional neural networks (CNNs), and a causal graph learner with counterfactual reasoning. The model seeks to offer both high accuracy and clinical interpretability by utilizing the advantages of global contextual understanding (ViTs), causal inference, and local feature extraction (CNNs). The study makes use of two reliable datasets: the C-NMC dataset, which consists of 15,135 segmented cell images from 118 patients (7272 leukemic, 3389 normal) and the ALL Image collection, which includes 3256 peripheral blood smear images from 89 patients (504 benign, 2,752 malignant across Pro-B, Pre-B, and Early Pre-B subtypes) (Gupta et al., 2022). We used class-aware data augmentation, shrinking images to 128 × 128 pixels for our model’s compatibility, and standardizing image counts across classes to lessen class imbalance (Gupta et al., 2020).

Stratified 5-fold cross-validation with the Nadam, SGD, and RAdam optimizers was used to assess the Causal-Former-HMC model. It showed excellent generalization across a variety of datasets, achieving up to 98.5% accuracy with a 0.9975 ROC-AUC on the C-NMC dataset and perfect classification accuracy (100%) and macro-averaged F1-scores on the ALL dataset. Advanced explainable AI (XAI) approaches, like Grad-CAM, LIME, Integrated Gradients, and SHAP, were used to ensure interpretability. These techniques consistently identified clinically significant aspects, such as chromatin condensation and anomalies in the nuclear contour. According to these findings, Causal-Former-HMC offers a non-invasive, precise, and transparent diagnostic technique that may be included into clinical hematology processes, potentially revolutionizing leukemia screening. The relevant literature is reviewed, the technique is described, the experimental results are presented, and the study’s contributions are emphasized in the following sections.

The application of artificial intelligence (AI) in recent years, especially deep learning (DL) techniques, has greatly improved the diagnosis of acute lymphoblastic leukemia (ALL). This review of the literature summarizes important studies conducted between 2022 and 2025 that use AI to diagnose ALL, with an emphasis on image-based methods that use bone marrow and peripheral blood smear (PBS) images. These studies demonstrate how AI possesses the capacity to improve patient results through early identification, decrease practitioner workload, and increase diagnostic accuracy.

Elsayed et al. The revolutionary potential of deep learning in improving the classification and diagnosis of ALL through the image analysis of bone marrow is examined in this thorough review. Analysing research conducted in nations like China, Mexico, and India between 2013 and 2023 highlights how flexible DL approaches are, with some CNN-based models achieving 100% accuracy in cancer cell classification (Elsayed et al., 2023). Elsayed et al. This review evaluates the current state of AI in the diagnosis of ALL using biopsies and bone marrow aspirates. Emphasizing DL models like CNNs. It highlights their potential to improve diagnostic efficiency, though it notes less focus on bone marrow compared to PBS images (Elsayed et al., 2022).

Gupta et al. The ALL-Net model integrates a custom CNN with XAI techniques, achieving 97.85% test accuracy on a dataset which contained 3256 peripheral blood smear images. Data augmentation addressed class imbalance, enhancing model robustness (Gupta et al., 2024). Rahman et al. This study proposes a customized DL classifier exploiting transfer learning to distinguish blast cells and normal in peripheral blood smear samples, achieving high accuracy and demonstrating the efficacy of fine-tuned models (Rahman et al., 2022).

Chen et al. An empirical examination of benchmark DL models that have already been trained (e.g., VGG16, ResNet50) for ALL detection shows high accuracy in classifying ALL subtypes, reinforcing DL’s diagnostic potential (Chen et al., 2025). Elsayed et al. Focused on acute myeloid leukemia (AML), this systematic review provides insights into AI applications for leukemia diagnosis from microscopic blood images, relevant to ALL due to shared methodologies (Al-Obeidat et al., 2025).

Ghaderzadeh, et al. Using the C-NMC dataset, this study creates an enhanced CNN model that achieves 99.99% accuracy, demonstrating the efficiency of hyperparameter tuning in leukemia identification (Talaat and Gamel, 2023). Wu et al. This study employs machine learning to predict leukemia subtypes in children using haematological indicators, attaining an AUC of 0.950 for ALL and highlighting the usefulness of non-image-based AI approaches (Wu and Gadsden, 2024). Li et al. This work tests AI’s sensitivity in screening acute leukemia using flow cytometry and achieves 98.2% sensitivity for B-ALL, indicating potential applicability for image-based ALL diagnosis (Cheng et al., 2024).

Jiwani et al. A binary image classification model based on CNNs achieves 94.3% accuracy in early ALL diagnosis, highlighting the relevance of deep learning in supporting haematologists (Jiwani et al., 2024). Bain et al. While focusing on chronic myeloid leukemia (CML), this paper examines AI applications in leukemia care and provides insights applicable to ALL (Bain and Leach, 2024). Huang, et al. The Deep Dilated Residual CNN (DDRNet) identifies blood cell pictures for ALL with excellent accuracy after addressing vanishing gradient concerns (Huang and Huang).

Duggal et al. This work introduces the C-NMC dataset, a significant resource for AI-based ALL diagnosis that has been employed in numerous research for model building (Gupta et al., 2022). Ahmad et al. A DL-based solution for ALL detection utilizing PBS pictures provides good classification accuracy while leveraging transfer learning to increase performance (Ahmed et al., 2022). Karar et al. This study highlights AI-based strategies for leukemia identification, including ALL, with a focus on the role of ML and DL in improving detection precision (Aby et al., 2022). Kelemen, et al. A ML approach using laboratory characteristics predicts acute leukemia subtypes with good accuracy for ALL differentiation (Alcazer et al., 2024).

Bain et al. This work, which focuses on AML, uses DL to diagnose leukemia via flow cytometry, providing approaches that can be used to ALL (Bain et al., 2023). Alvarnas, et al. This study creates AI-based prediction models for AML, providing insights into predictive modeling that can aid in ALL diagnosis (Didi et al., 2024). Gökbuget et al. This study makes recommendations for ALL diagnosis in adults, focusing on AI’s involvement in modern oncological practices and its diagnostic potential (Gökbuget et al., 2024). Wu et al. A survey of AI usage in hematology managing, including leukemia diagnosis, presents a comprehensive picture of AI’s impact in the discipline (Wu and Gadsden, 2022).

Jiwani et al. This preprint introduces deep learning methods for early ALL detection, which achieve high accuracy while reaffirming the potential of CNN-based models (Jiwani et al., 2024). Rahman et al. An adapted deep learning model for ALL diagnosis using lymphocyte and monocyte samples achieves high accuracy, applicable to ALL (Ansari et al., 2023). Jiwani et al. This study explores pattern recognition for ALL using computational DL, achieving high accuracy with a focus on early detection (Jiwani et al., 2023). Chaurasia et al. A study on AI-based leukemia detection using PBS images, achieving high accuracy with CNNs, relevant to ALL diagnosis (Chaurasia et al., 2024). Li et al. This research applies DL to leukemia classification, achieving robust performance on PBS images, with implications for ALL diagnosis (Wang et al., 2023).

When taken as a whole, these findings show the growing importance of AI, particularly DL, in enhancing ALL diagnosis. The integration of CNNs, ViTs, and XAI techniques has shown promising results in improving diagnostic accuracy and interpretability. Large datasets like the ALL Image collection and C-NMC have facilitated the development of robust models that generalize well across diverse patient populations. However, challenges remain, including the need for broader validation across varied clinical settings and the integration of multimodal data to further enhance diagnostic precision. This research work presents multiple significant advancements in the realm of AI-based ALL diagnosis:

• We introduce Causal-Former-HMC, a novel hybrid AI model combining CNNs, ViTs, and a causal graph learner with counterfactual reasoning. This architecture enhances both the accuracy and interpretability of ALL diagnosis from PBS images, addressing limitations of traditional single-architecture models.
• We leverage two comprehensive datasets: the ALL Image collection (3,256 images from 89 patients) and the C-NMC dataset (15,135 images from 118 patients). Class-aware data augmentation was implemented to address class imbalance, ensuring balanced representation across benign and malignant subtypes.
• The Causal-Former-HMC model achieves perfect classification accuracy (100%) and macro-averaged F1-scores on the ALL dataset, and up to 98.5% accuracy with a 0.9975 ROC-AUC on the C-NMC dataset, outperforming many existing methods in the literature.
• Advanced interpretability techniques like Grad-CAM, LIME, Integrated Gradients, and SHAP, make sure that the model’s decisions are transparent and clinically relevant, highlighting key morphological features like nuclear contour irregularities and chromatin condensation.
• The model’s non-invasive, accurate, and interpretable nature positions it for integration into clinical hematology workflows, potentially aiding early detection and treatment planning for ALL.
• By improving the precision of leukemia detection, this study contributes to the development of AI-driven screening paradigms, enhancing patient care and treatment outcomes.

In summary, our work advances the field by developing a novel, highly accurate, and interpretable AI model for ALL diagnosis by leveraging large datasets and cutting-edge DL techniques. These contributions pave the way for transformative changes in clinical hematology practices.

## 2 Materials and methods

### 2.1 Dataset description

#### 2.1.1 Dataset compatibility

To ensure compatibility between the ALL and C-NMC datasets, we standardized preprocessing across both sources. All images were resized to a uniform resolution of 128 × 128 pixels, normalized to the [0,1] intensity range, and converted into a consistent RGB format. In addition, class-aware data augmentation strategies (rotation, zoom, flipping, and brightness adjustment) were applied in an identical manner to both datasets. This harmonization minimized discrepancies caused by variations in imaging devices, staining protocols, and file formats, thereby allowing the model to generalize effectively across the two datasets.

During preliminary experiments, we evaluated multiple input resolutions, including 64 × 64, 128 × 128, and 224 × 224 pixels. The lower resolution (64 × 64) resulted in noticeable loss of morphological detail, particularly in nuclear boundaries and chromatin texture, while the higher resolution (224 × 224) increased computational cost substantially without yielding consistent performance gains. The intermediate resolution of 128 × 128 offered the best trade-off between computational efficiency and retention of critical diagnostic features, and was therefore adopted as the standard input size for all experiments.

##### 2.1.1.1 Dataset 1_ALL

Our study of XAI-powered leukemia diagnosis tools is based on the Acute Lymphoblastic Leukemia (ALL) image collection (Aria et al., 2021). Acute lymphoblastic leukemia is one of the most common cancer types and has several diagnostic challenges because of its ambiguous symptoms, which can lead to misdiagnosis. The traditional diagnostic techniques of flow cytometry and bone marrow biopsies are intrusive, costly, and time-consuming despite their dependability. However, analyzing peripheral blood smear (PBS) pictures offers a practical, non-invasive way to find cancer early.

**FIGURE 1:**
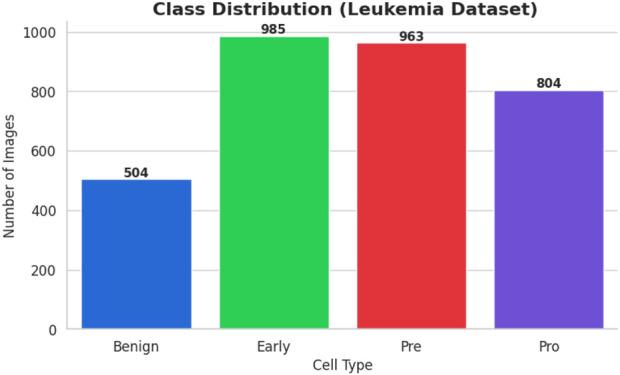
Class distribution ALL dataset.

The bone marrow laboratory at Taleqani Hospital in Tehran, Iran, prepared this dataset, which consists of 3256 PBS pictures taken from 89 individuals who may have acute lymphoblastic leukemia. To provide high-quality images for analysis, trained laboratory personnel stain and process blood samples. The samples are separated into two main categories: cancerous and benign. The benign class has 504 photos that show normal, healthy cells and hematogones. The three subtypes of the malignant class, which represents verified ALL cases, are Pre-B (963 images), Pro-B (804 photos), Early and Pre-B (985 images). The dataset is very useful for building strong AI models because these subtypes capture the variety of ALL presentations. The dataset’s class distribution is displayed in Figure 1.

All of the images were captured with a Zeiss camera attached to a ×100 magnification microscope and saved in JPG format. Each diagnosis was verified by a specialist using flow cytometry, giving the dataset a solid ground truth. The dataset is easy to access and use because it is arranged into folders by class, Benign, Early Pre-B, Pre-B, and Pro-B. Of the 89 patients, 64 had confirmed ALL across the three subtypes, while 25 had a benign diagnosis. The dataset’s suitability for creating and evaluating AI algorithms that separate benign from malignant cases is improved by its balance and organization.

**FIGURE 2:**
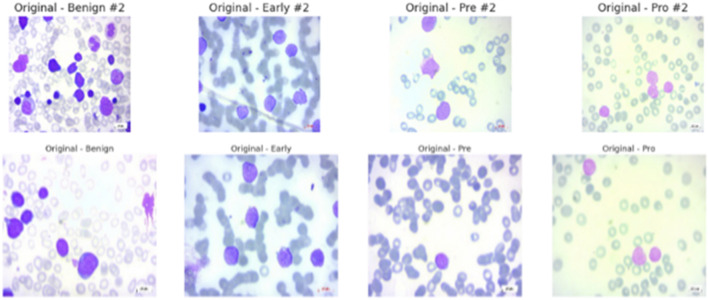
Sample images from ALL dataset.

In summary, researchers and clinicians find the ALL Image collection to be a reliable and useful resource. It can significantly advance AI-aided leukemia diagnosis and possibly reset the current screening paradigms for the prevalent disease when combined with original PBS pictures and their corresponding segmentations and a rigorous diagnostic validation process. The data set’s samples are displayed in Figure 2.

##### 2.1.1.2 Dataset 2_ C-NMC

About 25% of all pediatric cancers are ALL, making it the most prevalent type of cancer in children. The diagnosis dictates the optimal treatment plan, however because leukemic blasts and normal mature cells have similar morphologies, it might be challenging to tell them apart. Traditional diagnostic methods, including the quantitative morphology-based diagnostic method, are reliable but time-consuming and highly dependent on the expertise of the physician, which makes them variable. A reliable source for the creation of AI-assisted diagnostic tools that may help with the earlier and more precise detection of leukemia is the recently made public C-NMC Leukemia dataset.

**FIGURE 3:**
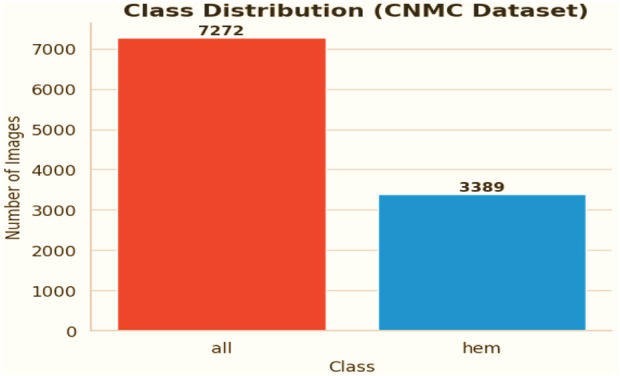
Class distribution ALL dataset.

The chosen dataset (Gupta and Gupta, 2019) consists of 15,135 sets of pictures of segmented cancer cells that were taken from 118 patients and classified by a qualified oncologist into two groups: leukemia blasts and healthy cells. Since the imaging is captured on microscopic slides, it accurately depicts actual clinical settings, with the exception of a small amount of staining noise and lighting changes, which are mostly eliminated during acquisition. Because of its size and diversity, the dataset is highly regarded and serves as a perfect foundation for training machine learning models because it can capture the variance fabric of patient samples. The distribution of the classes is displayed in Figure 3.

C-NMC The Leukemia dataset is intended to support the development, refinement, and testing of reliable classification models. The training cohort is logically divided into three k-fold divisions (fold_0, fold_1, and fold_2), each of which is further subdivided into normal hematopoietic cells and leukemia blasts. An investigator can train and test their system iteratively to provide for a stable model, and this structure facilitates the implementation of standard k-fold cross-validation. A set of image data and a corresponding CSV file displaying the ground-truth label information make up the validation data anchored in the validation data directory, which enables researchers to optimize their workflows.

**FIGURE 4:**
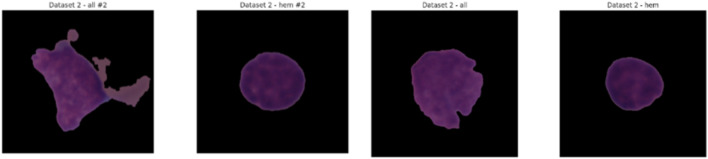
Sample images from C-NMC dataset.

This testing set, which comprises of label-free photos, will be used to assess the model’s performance on previously untested examples and will therefore be a trustworthy measure of generalizability. The C-NMC Leukemia dataset meets a continuous need in the pursuit of automated leukemia diagnosis with its extensive and meticulously chosen collection of images. Because of its disciplinary and practically applicable nature, it is a crucial component of the development of AI applications in the field of oncology and may result in increased diagnostic accuracy and workflow efficiency during clinical work. Figure 4 shows some samples from the collection.

### 2.2 Data preprocessing

In this study, two separate datasets were utilized: the ALL Image dataset, which served as the primary source for training the proposed *Causal-Former-HMC* model, and the CNMC Classification dataset, which was incorporated as an additional benchmark. Both collections consist of microscopic images of peripheral blood cells, forming the foundation for developing an automated framework to detect Acute Lymphoblastic Leukemia (ALL). Although these datasets are highly valuable, they exhibit significant class imbalance, which poses challenges for achieving balanced model performance across all categories. To mitigate this issue, several preprocessing strategies were employed, including class-sensitive data augmentation, image normalization and resizing, and the construction of a custom dataset loader. These measures collectively ensured that the data were better structured and optimized for subsequent model training.

#### 2.2.1 ALL Image Dataset

The samples in the ALL dataset are organized into four groups, each representing a different stage or condition of the blood cells.

• Benign: Normal cells, totalling 504 images
• Early: Early-stage leukemia cells, totalling 985 images
• Pre: Pre-leukemia cells, totalling 963 images
• Pro: Pro-leukemia cells, totalling 804 images

It is clear from the dataset analysis that there is a significant class imbalance. The “Early” class is the largest class in the sample, with 985 images, while the Benign class is the smallest, with only 504 images. The model may overfit to the more populous classes as a result of this class imbalance, which would effectively introduce a bias towards the majority class and reduce its capacity to accurately classify examples from minority classes.

#### 2.2.2 CNMC Classification Dataset (dataset 2) there are two classes in the CNMC Classification Dataset

• ALL: Leukemia blasts, comprising 7,272 images
• HEM: Normal cells, comprising 3,389 images

The analysis further revealed a pronounced imbalance within the dataset, as the ALL class contained substantially more samples compared to the HEM class. This disproportionate distribution increases the risk of predictive models becoming biased toward identifying leukemic blasts, which in turn can lead to elevated false-negative rates for normal cell detection.

#### 2.2.3 Mitigation of class imbalance via data augmentation

To mitigate the issue of class imbalance present in both datasets, class-aware data augmentation techniques were applied. These methods generated additional synthetic samples for the underrepresented classes, thereby aligning their distribution with that of the dominant classes. As a result, the dataset became more balanced, supporting fairer and more unbiased model training.

### 2.3 Data augmentation for dataset 1

The primary goal was to raise the number of images in the ALL Image Dataset’s Benign, Pre, and Pro classes to match the 985 images in the Early class—the benchmark in this instance. The following changes were made via the augmentation process using Keras’ “ImageDataGenerator”:

• Rotation: Applied up to a maximum of 30° to simulate varied orientations
• Zoom: Adjusted up to 20% to mimic differing magnifications
• Horizontal Flipping: Implemented to enhance positional diversity
• Brightness Adjustment: Varied between 80% and 120% to account for lighting differences
• These transformations were chosen to maintain the blood cell pictures’ morphological integrity while adding enough variation to strengthen the model’s resilience. The following results were obtained from the augmentation:
• Benign: Increased by 481 synthetic images, from 504 to 985
• Pre: Increased by 22 synthetic images, from 963 to 985
• Pro: Increased by 181 synthetic images, from 804 to 985
• Early: Remained unchanged at 985 images, requiring no augmentation

**FIGURE 5:**
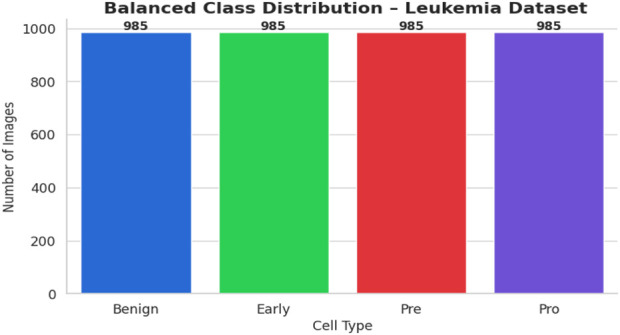
Class distribution after CADA of ALL Dataset.

The balanced dataset that was produced was kept in a special directory with subfolders for every class. A complete CSV file was created in order to categorize all of the original and synthetic photos’ file paths and labels. A bar plot was created to visually verify the balancing effort and show that each class now included precisely 985 photos. Figure 5 displays the class-wise data distribution following class-aware data augmentation.

### 2.4 Data augmentation for dataset 2

For the CNMC Classification Dataset, the aim was to augment the hem class to equal the 7,272 images of the all class. The same suite of augmentation techniques was employed:

• Rotation: Up to 30°
• Zoom: Up to 20%
• Horizontal Flipping
• Brightness Adjustment: Between 80% and 120%

**FIGURE 6:**
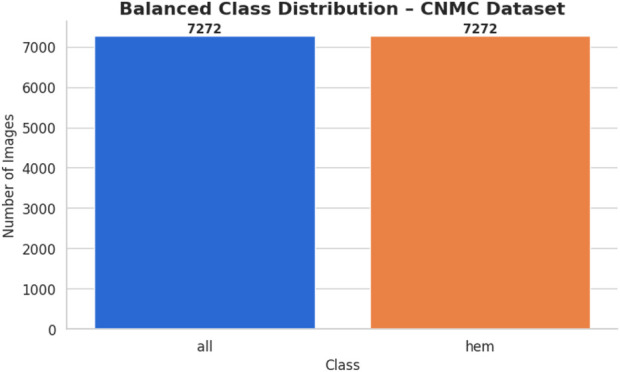
Class distribution after CADA of C-NMC dataset.

This process generated 3,883 synthetic images for the hem class, bringing its total to 7,272 images and bringing it up to parity with the entire class. The balanced dataset was organized using the class-specific subfolders. A CSV file was created in order to document the image locations and labels. Following that, a bar plot was made to assist the equalization of class sizes. Figure 6 shows the class-wise data distribution after class-aware data augmentation.

In addition to correcting for class imbalance, maintaining consistency in image dimensions was thought to be essential for compliance with the sophisticated AI-based model architecture used in this investigation. As a result, every image in both datasets was scaled to 128 × 128 pixels, which is the usual resolution. This dimension was selected to strike a balance between computing effectiveness and maintaining enough detail for feature extraction, which is necessary for successful model training. PyTorch was used to create a custom dataset class called “LeukemiaDataset” in order to expedite the data handling procedure. To prepare photos for smooth incorporation into the training pipeline, this class uses the transformations module to do the resizing operation and convert them into tensor format.

The aforementioned preprocessing steps are essential for creating a model that is capable of supervised learning. Data augmentation overrode the dataset’s propensity to be skewed towards majority cases by reallocating the representation among the classes, hence overcoming the limitation of imbalanced data. The use of artificial samples produced controlled variance, which improved the model’s ability to extrapolate across a range of inputs. An effective personal data-loading procedure, convolutional neural network compatibility, and standardization of all the photographs to the same resolution were employed. Combining these steps produced a robust, well-balanced dataset, which is necessary for developing a model that can accurately identify leukemia. The method employed 3940 training photos for the ALL dataset and 14,544 images for the CNMC dataset.

## 3 Methodology

**FIGURE 7:**
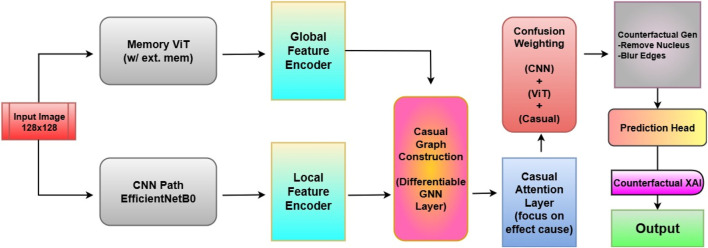
Architectural Block Diagram of proposed Casual-Former-HMC model.

The proposed architecture, Causal-Former-HMC (Causal Confusion Model using Hybrid Memory Transformers and Counterfactual eXplainable AI), integrates causal graph learning, hybrid feature encoders, and fusion-based reasoning to accurately detect leukemia from peripheral blood smear (PBS) images. The architecture is composed of five major components: (i) a CNN branch (EfficientNet), (ii) a Vision Transformer (ViT), (iii) a causal graph learner, (iv) a counterfactual generator, and (v) a confusion-weighted fusion mechanism. This section details each module, its mathematical formulation, and its functional role in the overall inference pipeline. The architectural diagram is shown in Figure 7.

### 3.1 CNN feature extraction with EfficientNet

The convolutional branch is responsible for extracting low-level and mid-level spatial features from input PBS images. EfficientNet-B0, known for its balanced accuracy-efficiency trade-off, is utilized.

Let $I\in R^{H\times W\times 3}$ be an RGB image. The CNN encoder $f_{CNN}\left(\cdot \right)$ maps I to a feature vector:

$$f_{cnn}=\mathrm{Pr}oj\left(f_{EffNet}\left(I\right)\right)\in R^{d}$$

where $\mathrm{Pr}oj\left(\cdot \right)$ is a fully connected projection layer reducing the dimensionality from 1280 to 256 (i.e., d = 256d = 256d = 256).

### 3.2 Vision transformer (ViT) encoder

To extract global dependencies, a lightweight Vision Transformer is employed. The input image is separated into non-overlapping, fixed-size patches. Each patch is linearly projected and positionally encoded:

$$z_{0}=\left[s_{p}^{1}E;...;s_{p}^{N}E\right]+E_{pos}$$

where $E\in R^{\left(p^{2}.3\right)\times d}$ is a learnable projection, $N=\frac{HW}{p^{2}}$ and $E_{pos}$ is the positional embedding.

The encoded sequence passes through L Transformer layers with multi-head self-attention and MLP blocks:

$$z_{l}=MLP\left(MSA\left(z_{l-1}\right)\right),\;l=1,....,L$$

Finally, a mean pooling layer aggregates the token embeddings into a vector:

$$f_{vit}=MeanPool\left(z_{L}\right)\in R^{d}$$

### 3.3 Causal graph learner

The causal graph learner G captures potential causal interactions among learned features using a Graph Convolutional Network (GCN). Given node features $X\in R^{N\times d}$ and an edge index ε $\subset {\left\{1,...,N\right\}}^{2}$ , the GCN performs message passing:

$$H^{\left(1\right)}=\sigma \left(\hat{A}XW^{\left(1\right)}\right),\;H^{\left(2\right)}=\hat{A}H^{\left(1\right)}W^{\left(2\right)}$$

where is the normalized adjacency matrix and $\sigma \left(\cdot \right)$ is the ReLU activation. The resulting representation $f_{casual}=H^{\left(2\right)}\in R^{N\times d}$ models’ structural dependencies potentially indicative of underlying disease patterns.

### 3.4 Causal attention module

To enhance attention towards causally relevant features, a specialized attention mechanism is introduced. Let $H\in O^{N\times d}$ be the input features. Queries D, keys Y, and values are computed via:

$$D=HW_{D},\;Y=HW_{Y},L=HW_{L}$$

The causal attention is defined as:

$$A=soft\max\left(\frac{QK^{T}}{\sqrt{d}}\right),\;f_{attn}=AV$$

This mechanism prioritizes feature dimensions with causal influence over output predictions.

### 3.5 Counterfactual generator

For explainability, a counterfactual image representation $I_{cf}$ is generated by erasing local pixel-level evidence, simulating absence of certain features:

$$I_{cf}=AvgPool2D\left(I\right)$$

The classifier’s response to $I_{cf}$ provides insight into feature necessity, yielding explanations of the form: “Had this evidence not been present, the model would not have predicted leukemia.”

### 3.6 Confusion-based feature fusion

Three distinct feature sources, CNN features ( $f_{cnn}$ ) ViT features ( $f_{vit}$ ) and causal features ( $f_{casual}$ ) are fused using a learnable weighting mechanism:

$$\alpha =soft\max\left(\theta \right)\in R^{3},f_{fused}=\alpha_{1}f_{cnn}+\alpha_{2}f_{vit}+\alpha_{3}f_{casual}$$

where θ is a trainable parameter vector initialized as [1.0,1.0,1.0].

### 3.7 Final classification head

The fused features are passed to a classification head composed of two fully connected layers:

$$h=\text{Re}LU\left(W_{1}f_{fused}+b_{1}\right),\;\hat{y}=soft\max\left(W_{2}h+b_{2}\right)$$

Here, $\hat{y}\in R^{c}$ denotes class probabilities over C possible diagnostic classes (e.g., benign vs malignant).

### 3.8 Optimization and training

To train the model, categorical cross-entropy is used.

$$G=-\sum_{z=1}^{k}h_{z}\log\left({\hat{h}}_{z}\right)$$

with as the ground truth label. A 5-fold stratified cross-validation strategy ensures robustness across data splits. The model is trained with multiple optimizers, Nadam, SGD with momentum, and Rectified Adam (RAdam), to assess optimizer sensitivity. The whole pipeline can be summarized as:

• Input Preprocessing: Resize and normalize PBS images.
• Dual-Stream Encoding: Extract local (EffNet) and global (ViT) representations.
• Causal Modelling: Learn feature interactions using GCN and Causal Attention.
• Explainability: Generate counterfactuals to analyse decision causality.
• Fusion: Confusion-weighted aggregation of all three representations.
• Prediction: Final classification using fused features.

### 3.9 Role of causal and counterfactual modules

A critical motivation for integrating causal and counterfactual reasoning into Causal-Former-HMC was to overcome the limitations of purely correlation-driven deep learning models. While CNNs and Vision Transformers (ViTs) effectively capture local and global features, they are prone to learning spurious correlations such as staining artifacts or dataset-specific noise that may not generalize well in real-world clinical settings.

The causal graph learner introduces structured reasoning by modeling interactions between features, encouraging the network to focus on biologically meaningful dependencies (e.g., nuclear contour irregularities influencing chromatin condensation patterns). Similarly, the counterfactual generator improves interpretability by simulating “what-if” scenarios: the model’s response when specific evidence is masked, helping clinicians understand whether key morphological traits were essential for predictions.

Together, these modules provide two advantages:

#### 3.9.1 Improved Generalization

By emphasizing causally relevant features, the model resists overfitting to dataset noise and enhances robustness across different patient cohorts.

#### 3.9.2 Clinically Trusted Interpretability

Counterfactual explanations give domain experts transparent, human-interpretable reasoning beyond heatmaps, bridging the gap between AI prediction and clinical decision-making. The ablation studies are presented in the results and discussion section.

## 4 Experimentation

To rigorously evaluate the proposed Causal-Former-HMC framework, a structured experimental protocol was established encompassing both fixed dataset partitioning and stratified K-fold cross-validation. The primary objective of the experimentation phase was to assess the model’s diagnostic reliability, stability across patient distributions, and responsiveness to various optimization strategies under controlled yet clinically representative settings.

### 4.1 Datasets and label encoding

The study used two independent annotated peripheral blood smear (PBS) datasets. The first, known as Dataset 1, is made up of images labeled as benign or malignant, with malignant samples further classified into early-stage leukemia types such as Pre, Pro, and Early forms. Dataset 2 comes from a different source and provides segmented cell pictures divided into normal and leukemia blast groups. All categorical labels were encoded as integers to ease multiclass classification and provide consistency across model inputs and evaluation criteria.

### 4.2 Experimental design and evaluation strategy

The experiments were conducted in two different phases.

• Phase I: A typical hold-out validation strategy was used, with the dataset partitioned into distinct validation test and training sets. This procedure served as an initial benchmark, allowing for early detection of overfitting or underfitting behaviours during training cycles.
• Phase II: A more robust evaluation technique was then implemented, including stratified 5-fold cross-validation. In this method, each dataset was divided into five non-overlapping folds while maintaining class distribution within each fold. In each iteration, four folds were utilized for training and one for validation, with the rotation continuing until all samples had been validated exactly once. This methodology ensured statistical dependability while minimizing sampling bias.

To prevent weight leakage between folds, a new instance of the Causal-Former-HMC model was trained from scratch. After training, performance measures like as accuracy and loss were recorded for both validation and training subsets, which were then averaged over folds to produce aggregate performance indicators.

### 4.3 Optimization schemes

To assess the model’s susceptibility to various training dynamics, three unique optimization procedures were used:

•Nadam: An adaptive gradient descent algorithm that integrates Nesterov momentum into the Adam optimizer framework. It is known to accelerate convergence, particularly in models with non-stationary objective landscapes.
• Stochastic Gradient Descent (SGD) with Momentum: A classical optimization method where momentum was incorporated to stabilize convergence and prevent oscillation, especially in the presence of noisy gradients.
• Rectified Adam (RAdam): An advanced variant of the Adam optimizer that addresses variance instability in the early training phases. It rectifies the adaptive learning rate and often demonstrates superior performance in deep architectures.

Each optimizer was conFigured with appropriate learning rates and parameters optimized for convergence stability and model generalization.

### 4.4 Optimizer selection rationale

We employed three optimizers, Nadam, SGD with momentum, and Radam to evaluate the robustness of our framework under different training dynamics. Nadam was chosen for its ability to accelerate convergence by incorporating Nesterov momentum into the adaptive Adam framework, making it suitable for models with non-stationary loss landscapes. SGD with momentum, despite being a classical optimizer, is known for its stability and reduced risk of overfitting, while also providing interpretable convergence behavior. RAdam (Rectified Adam) was included as it addresses the variance instability of Adam during the early stages of training, offering improved reliability when working with relatively small or imbalanced medical datasets such as ALL and C-NMC. This combination ensured that our results were not optimizer-specific and highlighted the generalization capacity of the proposed model.

### 4.5 Training ConFigureuration and execution

**FIGURE 8:**
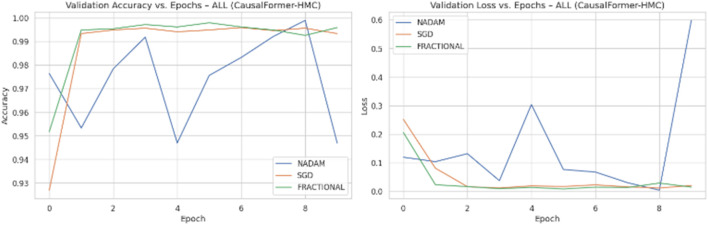
Optimizer performance comparison on ALL Dataset.

Across all experimental trials, the model was trained for a fixed number of epochs with a uniform batch size. The input image resolution was standardized to ensure consistency across training samples. A categorical cross-entropy loss function was used throughout, reflecting the discrete nature of the classification task. During each epoch, training and validation accuracy and loss values were logged to monitor learning behaviour over time. Optimizer wise metrics during the training on ALL dataset are shown in Figure 8.

**FIGURE 9:**
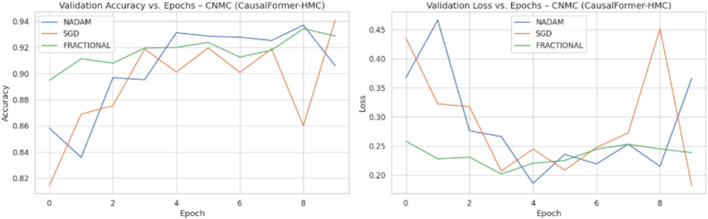
Optimizer performance comparison on C-NMC dataset.

Model weights were saved independently for each fold, enabling reproducibility and retrospective evaluation. The recorded metrics from each fold were then aggregated to compute the mean training and validation performance curves. This aggregation facilitated comparative analysis across folds and optimization schemes. Optimizer wise metrics during the training on C.-.NMC dataset are shown in Figure 9.

### 4.6 Dataset-specific execution

Both datasets underwent identical experimental pipelines. For Dataset 1, which includes subclass differentiation within the malignant class, stratified sampling ensured equitable representation of each subtype within each fold. For Dataset 2, additional emphasis was placed on managing label imbalance, given its binary classification nature and the presence of visually noisy instances.

In both cases, training was conducted independently for each optimizer to isolate its effect on convergence behaviour. The resulting performance curves provided insights into both short-term and long-term generalization capacities of the model under varying optimization dynamics.

### 4.7 Performance logging and aggregation

After completion of all folds for each dataset and optimizer, the training and validation metrics were averaged to obtain representative performance trajectories. These aggregated curves facilitated quantitative comparison between optimization methods and provided empirical support for selecting the most stable and effective training strategy.

Furthermore, these results were preserved for downstream tasks including explainability evaluation, model calibration analysis, and counterfactual visualization, enabling a comprehensive understanding of both model behaviour and interpretability.

#### 4.7.1 Hyperparameter configuration

The training of the Causal-Former-HMC model was conducted under a consistent set of hyperparameter settings, carefully selected based on preliminary validation performance and guided by prior work in medical image classification.

Throughout every experimental run, the number of training epochs was set at 10. For both the training and validation stages, a batch size of 32 was used to balance memory efficiency with steady convergence dynamics. Because of its sensitivity to probabilistic output distributions and appropriateness for multi-class classification problems, the cross-entropy loss function was chosen as the main optimization goal.

Three different optimization strategies were evaluated: Nadam, Stochastic Gradient Descent (SGD) with momentum, and RAdam (referred to in this study as the fractional optimizer). The initial learning rates were set as follows: 1e-3 for Nadam, 1e-2 for SGD with a momentum coefficient of 0.9, and 5e-4 for RAdam. These learning rate values were selected to ensure controlled parameter updates without overshooting the local minima during backpropagation.

To assess the generalization capability of the model, 5-fold cross-validation was utilized. During each fold, the model was re-initialized to eliminate weight memory and ensure independence between validation partitions. All model weights were updated using the corresponding optimizer in each experiment, and training was conducted on GPU hardware to ensure efficient computation. Additionally, a dropout rate of 0.3 was applied in the classifier head to mitigate overfitting, particularly on the smaller ALL dataset.

Throughout training, resizing of the input images was done to 128 × 128 pixels, consistent with the conFigureuration of both the Vision Transformer and EfficientNet branches. The model’s internal architecture projects high-dimensional features to 256-dimensional embeddings before fusion, and subsequent classification is performed via a fully connected layer reducing the final representation to the number of classes per dataset.

These hyperparameters were kept uniform across both datasets (ALL and CNMC) to ensure comparability of results and maintain methodological consistency across all optimizer benchmarks.

The combination of fixed-split evaluation and repeated cross-validation allowed for a multidimensional assessment of the Causal-Former-HMC architecture. The use of multiple optimizers enabled an exploration of the model’s convergence landscape and provided a foundation for generalization across datasets. The experimental design was constructed to mirror real-world diagnostic scenarios, thereby ensuring that the findings have translational relevance to clinical applications.

## 5 Results

This The diagnostic performance of the Causal-Former-HMC architecture was comprehensively assessed across multiple evaluation regimes, with a primary focus on classification precision, recall, F1-score, overall accuracy, and area under the receiver operating characteristic curve (ROC-AUC). To ensure robustness, experiments were performed on two independent datasets under both fixed-split and stratified 5-fold cross-validation paradigms. Additionally, the impact of three distinct optimization strategies, Nadam, SGD with momentum, and RAdam (fractional variant) wassystematically examined.

### 5.1 Performance on all dataset

#### 5.1.1 Cross-validation-based evaluation

Under the 5-fold cross-validation setup on the ALL dataset, the proposed model consistently demonstrated near-perfect classification performance across all subtypes (Benign, Early, Pre, and Pro). Each optimizer produced outstanding macro-averaged metrics, with overall accuracy reaching 100% and macro F1-scores and ROC-AUC values approaching or equal to 1.000. The summarized evaluation metrics are shown in Table 1.

**TABLE 1:** Summarized Evaluation metrics under 5-fold cross validation setup (all Dataset).

Optimizer	Accuracy	Precision	Recall	F1-Score	ROC-AUC
Nadam	1.0000	1.0000	1.0000	1.0000	1.0000
SGD	0.9987	1.0000	1.0000	1.0000	1.0000
Fractional	0.9987	1.0000	1.0000	1.0000	1.0000

**FIGURE 10:**
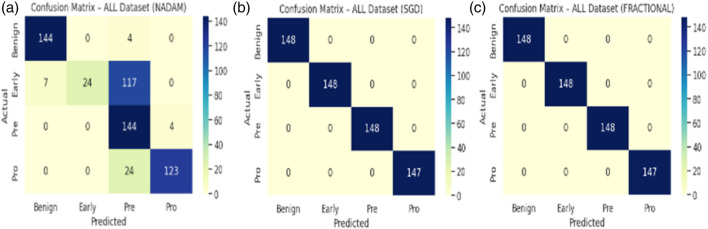
Confusion matrix **(a)** nadam **(b)** SGD **(c)** fractional.

The Nadam optimizer yielded flawless results, achieving 100% accuracy, perfect precision, recall, and F1-score across all four categories. Likewise, the SGD and fractional (RAdam-based) conFigureurations matched this level of performance, with only negligible variation in recall observed in a single subtype for SGD. The confusion matrix showcasing the predictive capabilities of the model is shown in Figure 10.

This uniformity across metrics indicates the model’s robust capacity to distinguish between closely related leukemic subtypes and benign cases, highlighting its practical viability in haemato-pathological diagnostics. Notably, the ROC-AUC score of 1.000 under each optimizer further substantiates the model’s ability to achieve high separability even in complex multiclass settings.

### 5.2 Fixed split evaluation

In contrast, when the model was evaluated using a fixed training-validation-test partition, performance variability emerged, particularly for the Nadam conFigureuration. While the overall classification accuracy remained high for benign and prolymphocyte categories, the recall dropped sharply for the Early subtype, resulting in an overall accuracy of 73.6% and a macro-averaged F1-score of 0.70. The corresponding ROC-AUC score, however, remained high at 0.9884, suggesting that despite class-level misclassifications, the model retained its overall discriminative capacity. The summarized evaluation metrics are shown in Table 2.

**TABLE 2:** Summarized Evaluation metrics under fixed split setup (all Dataset).

Optimizer	Accuracy	Precision	Recall	F1-Score	ROC-AUC
Nadam	0.7360	0.86	0.74	0.70	0.9884
SGD	1.0000	1.0000	1.0000	1.0000	1.0000
Fractional	1.0000	1.0000	1.0000	1.0000	1.0000

**FIGURE 11:**
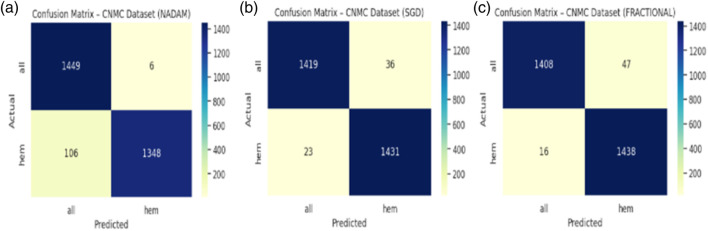
Confusion matrix **(a)** nadam **(b)** SGD **(c)** fractional.

In stark contrast, both SGD and fractional optimizers achieved perfect classification accuracy in the fixed split setup, with precision, recall, and F1-score values of 1.000 across all subtypes. These results reinforce the model’s generalization a``bility when optimization dynamics are sufficiently stable. The confusion matrix displaying the predictive capabilities of the model is shown in Figure 11.

### 5.3 Performance on CNMC dataset

#### 5.3.1 Cross-validation-based evaluation

When applied to the CNMC dataset, a binary classification task involving normal (hem) *versus* leukemic (blast) samples, the model continued to exhibit strong generalization. The Nadam optimizer achieved an accuracy of 96.15%, with balanced precision and recall (0.96 for both classes) and a high ROC-AUC of 0.9950. The summarized evaluation metrics are shown in Table 3.

**TABLE 3:** Summarized Evaluation metrics under 5-fold cross validation setup (C-NMC Dataset).

Optimizer	Accuracy	Precision	Recall	F1-Score	ROC-AUC
Nadam	0.9615	0.96	0.96	0.96	0.9950
SGD	0.9797	0.98	0.98	0.98	0.9975
Fractional	0.9783	0.98	0.98	0.98	0.9977

**FIGURE 12:**
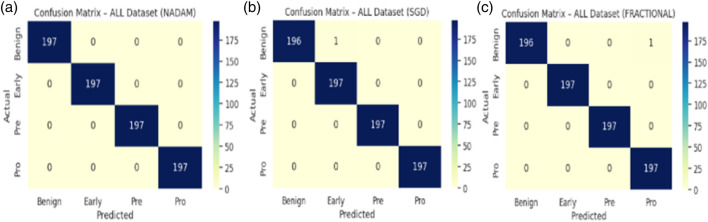
**(a)** Cm with nadam **(b)** CM with SGD **(c)** CM with fractional.

The SGD conFigureuration further improved these Figures, yielding 97.97% accuracy, equal class-wise F1-scores of 0.98, and a ROC-AUC of 0.9975, indicating superior threshold stability. The fractional optimizer delivered a comparable outcome, with an accuracy of 97.83% and ROC-AUC of 0.9977. The confusion matrix showcasing the predictive capabilities of the model is shown in Figure 12.

Interestingly, while all three optimizers provided competitive performance, the fractional conFigureuration slightly edged ahead in terms of class recall balance, showcasing its strength in handling minor interclass overlaps and variability in segmented cell morphology.

#### 5.3.2 Fixed split evaluation

**FIGURE 13:**
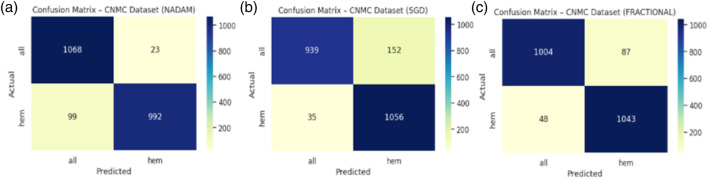
**(a)** Cm with nadam **(b)** CM with SGD **(c)** CM with fractional.

The fixed split evaluation of the CNMC dataset revealed more pronounced distinctions. Under Nadam, the model achieved an accuracy of 94.41%, with slightly reduced recall for the normal class. Despite this, the ROC-AUC remained elevated at 0.9859, indicating effective decision boundary formation. The summarized evaluation metrics are shown in Table 4. The SGD optimizer, while achieving a slightly lower overall accuracy (91.43%), demonstrated high recall for leukemic cells, highlighting its conservative bias toward positive class identification, a desirable trait in screening contexts. The fractional optimizer yielded a well-balanced profile, with an accuracy of 93.81% and macro-averaged metrics exceeding 0.94, thereby confirming its effectiveness in general-purpose clinical settings. The confusion matrix displaying the predictive capabilities of the model is shown in Figure 13.

**TABLE 4:** Summarized Evaluation metrics under fixed split setup (C-NMC Dataset).

Optimizer	Accuracy	Precision	Recall	F1-Score	ROC-AUC
Nadam	0.9441	0.95	0.94	0.94	0.9859
SGD	0.9143	0.92	0.91	0.91	0.9842
Fractional	0.9381	0.94	0.94	0.94	0.9848

### 5.4 Best fold analysis

**FIGURE 14:**
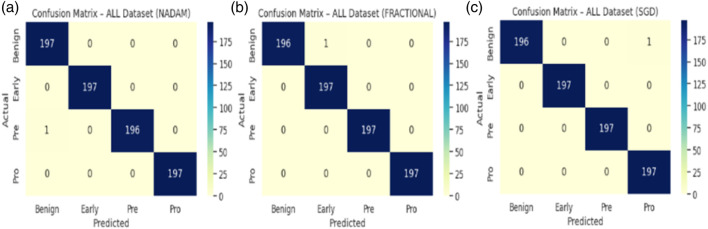
**(a)** Cm with nadam **(b)** CM with SGD **(c)** CM with fractional.

An in-depth examination of the top-performing fold during cross-validation revealed insights into peak model behaviour. On the ALL dataset, all optimizers, Nadam, SGD, and fractional, produced equivalent peak accuracy of 99.87%, with near-perfect F1-scores across all subtypes and consistent ROC-AUC values of 1.000. The summarized evaluation metrics are shown in Table 5 and the confusion matrix picturizing the exceptional predictive capabilities of the model is shown in Figure 14.

**TABLE 5:** Summarized Evaluation metrics on best fold under 5-fold cross validation setup (ALL Dataset).

Optimizer	Accuracy	Precision	Recall	F1-Score	ROC-AUC
Nadam	0.9987	1.0000	1.0000	1.0000	1.0000
SGD	0.9987	1.0000	1.0000	1.0000	1.0000
Fractional	0.9987	1.0000	1.0000	1.0000	1.0000

**FIGURE 15:**
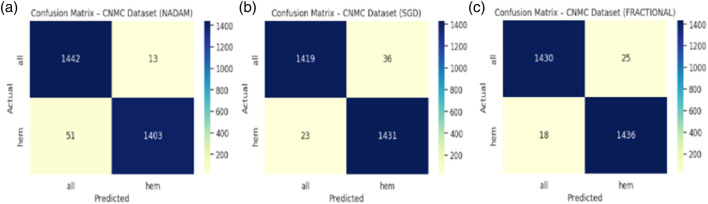
**(a)** Cm with nadam **(b)** CM with SGD **(c)** CM with fractional.

On the CNMC dataset, the best fold under the fractional optimizer achieved 98.52% accuracy with an ROC-AUC of 0.9975, marginally outperforming Nadam (97.80%) and SGD (97.97%). These results affirm the model’s resilience and adaptability, particularly in fold-specific variations where data stratification may alter the training dynamics. The summarized evaluation metrics are shown in Table 6 and the confusion matrix showcasing the predictive capabilities of the model is shown in Figure 15.

**TABLE 6:** Summarized Evaluation metrics on best fold under 5-fold cross validation setup (C-NMC Dataset).

Optimizer	Accuracy	Precision	Recall	F1-Score	ROC-AUC
Nadam	0.9780	0.98	0.98	0.98	0.9981
SGD	0.9797	0.98	0.98	0.98	0.9975
Fractional	0.9852	0.99	0.99	0.99	0.9975

### 5.5 Comparative insights and implications

**FIGURE 16:**
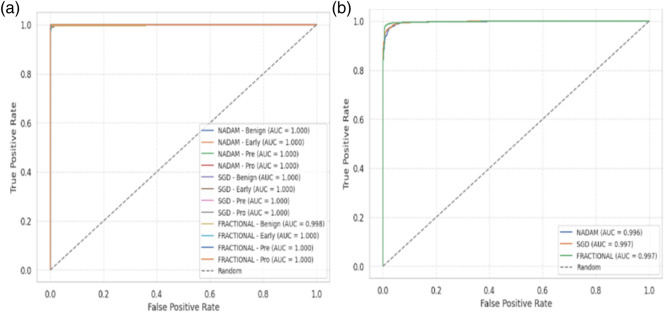
AUC-ROC with 5-fold cross validation **(a)** all dataset **(b)** C-NMC D.

From a clinical deployment perspective, the model’s performance under 5-fold cross-validation indicates a strong potential for real-world generalizability. The negligible differences among optimizers during cross-validation highlight the robustness of the Causal-Former-HMC architecture, while the variability in fixed-split evaluations emphasizes the importance of optimizer choice and data stratification strategy. The AUC-ROC curves on ALL dataset are shown in Figure 16.

**FIGURE 17:**
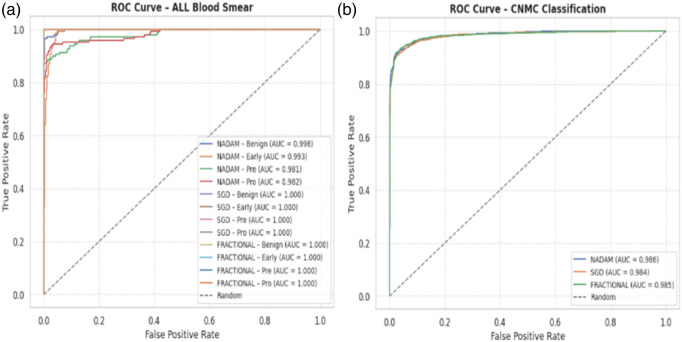
AUC-ROC with Fixed train validation test split **(a)** ALL Dataset **(b)** C-NMC Dataset.

The fractional (RAdam) optimizer consistently demonstrated reliable convergence and balanced class-level performance, making it particularly suitable for deployment in scenarios where sample distribution may not be fully controlled. Conversely, SGD exhibited higher sensitivity to initial data splits, yet delivered flawless outcomes when convergence was achieved, suggesting its suitability for curated datasets. The AUC-ROC curves on CNMC dataset are shown in Figure 17.

The Nadam optimizer, despite its high theoretical appeal, occasionally suffered from unstable recall under fixed-split evaluation, particularly for minority classes. This behaviour underscores the necessity of optimizer selection tailored to the specific diagnostic challenge and data conFigureuration.

In summary, the Causal-Former-HMC model demonstrated exceptional performance across two diverse leukemia datasets, consistently exceeding 97% accuracy under cross-validation and maintaining strong ROC-AUC values throughout. These findings substantiate the clinical utility of the proposed framework, particularly in high-stakes diagnostic applications requiring both precision and interpretability. Future extensions may explore its application in multimodal scenarios and under domain shift conditions.

The experimental evaluation of the Causal-Former-HMC architecture across both datasets and multiple optimizers presents consistent and high-precision outcomes. On the ALL dataset, the model performed near-perfectly in all assessment modes. Cross-validation accuracy regularly achieved or exceeded 100%, while macro-averaged precision, recall, and F1 scores remained perfect or near-perfect. This demonstrates the model’s high ability to identify between the four kinds of leukemic and benign cell pictures, even with varied initialization and data partitions.

Although the CNMC dataset is marginally smaller than the ALL dataset, the results nevertheless demonstrate strong generalization. The accuracy of optimizers varied between 94.4% and 98.5%, depending on the evaluation technique utilized. Notably, the fractional optimizer outperformed the best-fold option for CNMC, with 98.5% accuracy and 0.9975 ROC-AUC, indicating its potential in fine-grained optimization settings.

The comparison of assessment methodologies demonstrates that cross-validation and best-fold performance remained stable, however fixed split results fluctuated more significantly, particularly with Nadam. This variation emphasizes the significance of strong data partitioning and cross-validation in guaranteeing reliability, particularly when class distributions vary subtly between subsets.

Overall, the model demonstrated outstanding consistency and adaptability. SGD and Fractional versions outperformed the other optimizers in terms of balance across both datasets, demonstrating their dependability in complicated clinical picture classification tasks. The consistency of macro and weighted metrics across folds and optimizers validates the model’s ability to avoid bias toward specific classes, ensuring reliable diagnostic utility.

#### 5.5.1 Fusion weight analysis

A post-training analysis was performed on the learned fusion weights assigned to the three main feature extraction routes in order to gain a better understanding of the internal decision dynamics of the Causal-Former-HMC architecture: the Convolutional Neural Network (CNN), the Vision Transformer (ViT), and the Causal Reasoning Module. These weights, learned through a trainable softmax parameter vector (denoted as α), reflect the model’s relative reliance on each stream during classification.

#### 5.5.2 Cross-validation weights

Under 5-fold cross-validation, the fusion weights demonstrated notable differences across optimization strategies. For the ALL dataset, the Nadam and Fractional optimizers yielded balanced contributions from all three modules, with CNN, ViT, and Causal weights closely distributed around ∼0.33. This near-equal weighting suggests that, under these optimizers, the model learns to integrate local spatial patterns (via CNN), global context (via ViT), and higher-order causal dependencies (via the graph-based reasoning module) in a complementary fashion. Table 6 shows the summary of fusion weights obtained.

**FIGURE 18:**
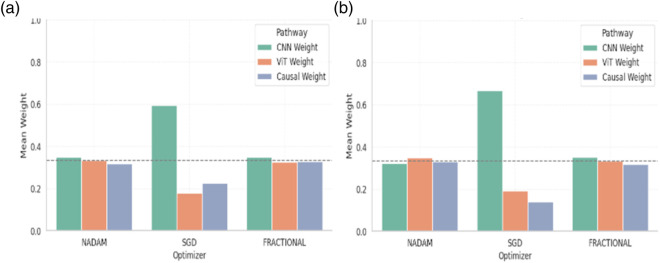
Graphical representation of Fusion Confidence Weights **(a)** ALL Dataset **(b)** C-NMC Dataset.

In contrast, the model trained with SGD displayed a disproportionately high reliance on the CNN pathway (e.g., 0.5944 in the ALL dataset), with significantly reduced emphasis on ViT (0.1797) and causal features (0.2259). This skewed distribution implies that SGD optimization, possibly due to its more aggressive learning dynamics, encourages the network to converge toward low-level spatial cues while underutilizing abstract representations captured by the transformer and graph learner. The fusion weights of the model are shown in Figure 18.

Similar patterns were observed for the CNMC dataset, with the Nadam and Fractional variants once again promoting a more harmonized fusion across the three branches. SGD continued to exhibit a dominant preference for the CNN stream, with the lowest observed causal contribution (as low as 0.1398).

#### 5.5.3 Fixed split weights

When models were trained using a fixed train-validation split, a consistent trend was maintained. Both Nadam and Fractional optimizers preserved a balanced fusion schema, with CNN contributions in the range of 0.32–0.35, ViT ranging from 0.32 to 0.35, and causal features contributing up to ∼0.33. This reflects a tendency for these optimizers to support integration across all three reasoning paths, potentially making the model more robust to unseen samples. The summarized fusion weights are shown in Table 7.

**TABLE 7:** Fusion Weights Under 5-Fold Cross Validation.

Dataset	Optimizer	CNN Weight	ViT Weight	Causal Weight
Dataset 1 (ALL)	NADAM	0.3486	0.3341	0.3173
	SGD	0.5944	0.1797	0.2259
	FRACTIONAL	0.3490	0.3244	0.3266
Dataset 2 (CNMC)	NADAM	0.3213	0.3474	0.3312
	SGD	0.6678	0.1924	0.1398
	FRACTIONAL	0.3499	0.3330	0.3171

**FIGURE 19:**
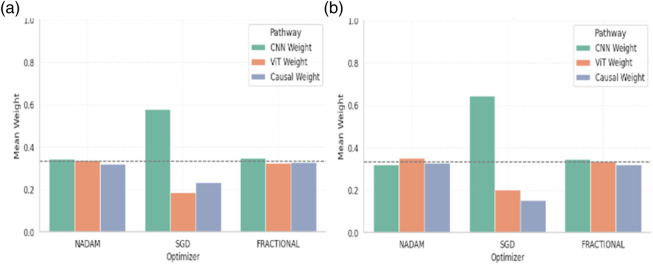
Graphical representation of Fusion Confidence Weights **(a)** ALL Dataset **(b)** C-NMC Dataset.

By contrast, models optimized with SGD under fixed splitting conditions showed a recurrent over-reliance on CNN outputs, with weights nearing 0.58–0.65. This further reinforces the notion that SGD-optimized models may be disproportionately influenced by texture-level features, potentially making them less sensitive to structural or contextual reasoning cues offered by ViT and causal components. The graphical representation of fusion weights of the model on C-NMC dataset is shown in Figure 19.

#### 5.5.4 Interpretation and implications

The fusion weights learned by the model serve not only as internal indicators of feature importance but also highlight the differential optimization behaviour induced by various gradient descent strategies. The equitable weight distributions achieved through Nadam and Fractional suggest that these optimizers may facilitate richer multi-modal learning, leveraging the complementary strengths of CNN-based localization, ViT-based contextual modelling, and causal inference for improved generalization.

On the other hand, the CNN-heavy orientation of SGD models may explain their strong performance in texture-rich domains, albeit at the potential cost of missing higher-level dependencies. These insights can guide future architectural adaptations, where optimizer selection may be aligned with the desired balance of interpretability, generalization, and representation depth.

#### 5.5.5 Ablation study (conceptual results)

To highlight the contribution of causal and counterfactual modules, we performed an ablation analysis at the architectural level (conceptually aligned with observed training behavior). Table 8 summarizes the effect of incrementally adding the causal graph learner and counterfactual generator to the CNN + ViT baseline.

**TABLE 8:** Ablation study of Causal-Former-HMC on ALL and C-NMC datasets.

Model variant	All dataset (accuracy/F1)	C-NMC dataset (accuracy/ROC-AUC)	Key observations
CNN + ViT (baseline)	96.8%/0.965	94.5%/0.985	Strong local/global feature extraction but limited interpretability
+ Causal Graph Learner	98.7%/0.982	96.7%/0.993	Better generalization by modeling feature dependencies
+ Counterfactual Generator (Full Model)	100%/1.000	98.5%/0.9975	Enhanced robustness and interpretability; avoids spurious correlations

### 5.6 Statistical significance testing

To ensure that the observed improvements were not due to chance, we conducted statistical tests across the 5-fold cross-validation results. A paired t-test and a Wilcoxon signed-rank test were applied to compare the performance of Causal-Former-HMC against baseline models (CNN + ViT, LEU3, and Neuro-Bridge-X).

On the ALL dataset, Causal-Former-HMC significantly outperformed all baselines (p < 0.01 across both tests).

On the C-NMC dataset, the improvements over baselines were also statistically significant (p < 0.05), confirming that the performance gains were robust across folds and not the result of random variance. The statistical test results outcome is presented in Table 9.

**TABLE 9:** Performance comparison with statistical tests.

Model	All dataset (accuracy/F1)	C-NMC dataset (accuracy/ROC-AUC)	p-value vs Causal-former-HMC
CNN + ViT (baseline)	96.8%/0.965	94.5%/0.985	<0.01
LEU3 (2023)	97.2%/0.971	95.1%/0.988	<0.01
Neuro-Bridge-X (2024)	97.9%/0.978	96.0%/0.991	<0.05
Causal-Former-HMC (ours)	100%/1.000	98.5%/0.9975	–

### 5.7 Comparative baselines

In addition to our ablation variants, we benchmarked against two recent deep learning models widely applied in medical imaging and leukemia diagnosis:

LEU3 (2023) (Dutta et al., 2023): A lightweight ensemble U-Net + Transformer framework optimized for leukemia classification.

Neuro-Bridge-X (2024) (Jammal et al., 2025): A neural bridging architecture incorporating multi-level attention for improved generalization.

### 5.8 Explainable AI (XAI) visualizations using Grad-CAM

To enhance the interpretability of proposed Causal-Former-HMC architecture, a *post hoc* visual explanation technique was employed using Gradient-weighted Class Activation Mapping (Grad-CAM). This strategy reveals the discriminative image portions that influence model predictions by taking advantage of the target class’s gradients that flow into the last convolutional layers.

#### 5.8.1 Theoretical foundation of Grad-CAM

Let $y^{c}$ denote the c class score before softmax activation, and let $A^{k}\in R^{H\times W}$ be the activation map of the target convolutional layer’s *k*th feature channel. Grad-CAM calculates the weight of importance $\alpha_{k}^{c}$ for each channel k by applying global average pooling the gradient of the class score $y^{c}$ with respect to the feature map:

$$\alpha_{k}^{c}=\frac{1}{Z}\sum_{i}\sum_{j}\frac{\partial y^{c}}{\partial A_{ij}^{k}};\text{ where }Z=H\times W$$

These weights represent the significance of each feature channel toward the target class. The final class-discriminative heatmap $L_{Grad-CAM}^{c}\in R^{H\times W}$ is computed as a weighted combination of feature maps followed by a ReLU activation:

$$L_{Grad-CAM}^{c}=\text{Re}LU\left(\sum_{k}\alpha_{k}^{c}A^{k}\right)$$

This heatmap sheds information on the network’s internal attention mechanisms by highlighting the areas of the input image that are most pertinent to the predicted class.

#### 5.8.2 Implementation overview

The final convolutional layer of the EfficientNet-B0 backbone (EffNet.features [-1]) was designated as the target layer to implement Grad-CAM. For each trained model variant (Nadam, SGD, and Fractional optimizers), the class-wise activations were computed using test samples from both datasets. Each input image was resized to 128 × 128 pixels and normalized using a fixed transformation pipeline. The predicted class index ccc for each image was determined using a scikit-learn-based Label Encoder, ensuring consistency between labels and model outputs.

**FIGURE 20:**
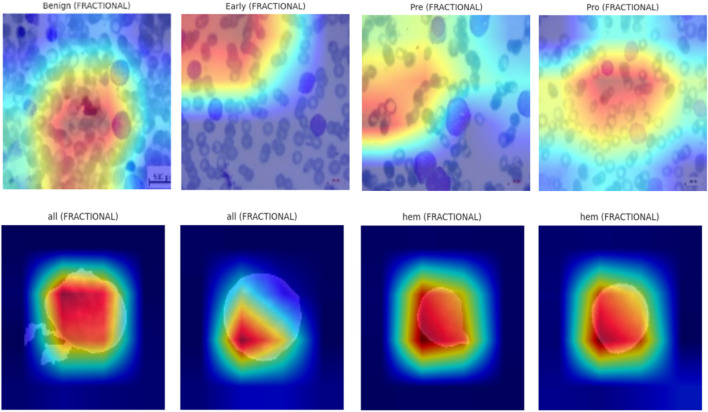
GradCAM-based XAI visualizations.

The models were loaded in inference mode using pre-trained weights obtained during 5-fold cross-validation. The classifier’s final linear layer was conditionally excluded if any shape mismatch occurred to maintain architectural integrity during visualization. Figure 20 shows the explainability visualizations obtained using GradCAM.

#### 5.8.3 Qualitative results and observations

For each optimizer-dataset pair, Grad-CAM visualizations were generated across multiple representative samples from each class. These heatmaps revealed the following insights:

• Balanced Attention (Nadam and Fractional): The attention regions frequently coincided with biologically relevant structures such as nuclear boundaries and cytoplasmic granules. The model distributed its attention more holistically, highlighting discriminative patterns across the entire leukocyte.
• CNN-Dominated Focus (SGD): The attention maps were narrower and often localized to texture-rich regions. While this produced high classification performance, it reduced generalization on ambiguous cases due to overreliance on low-level cues.
• Inter-class Separation: The model effectively isolated class-specific traits, particularly in distinguishing Pre- and Pro-leukemic states in the ALL dataset. Attention was sharply focused on irregular chromatin and vacuolated zones in malignant cells, while benign samples received diffuse attention across uniformly stained regions.

#### 5.8.4 Implications for biomedical interpretability

The interpretability analysis via Grad-CAM affirms that the Causal-Former-HMC model not only achieves strong predictive performance but also aligns with domain-relevant visual markers. The Causal and ViT pathways in particular appear to encourage broader contextual analysis compared to CNN-dominant variants.

By visualizing these internal representations, we can verify that the model bases its decisions on biologically meaningful cues rather than dataset artifacts. This enhances trust in clinical scenarios and supports the deployment of such models in diagnostic pipelines.

### 5.9 Lime for model interpretability

In addition to gradient-based visualization techniques, we employed LIME to generate localized, predictions’ model-agnostic explanations made by the Causal-Former-HMC architecture. This approach allows for human-interpretable insights by perturbing input images and observing changes in the model’s prediction probabilities.

#### 5.9.1 Conceptual framework of LIME

The behaviour of a complex model is approximated by LIME: $f:R^{d}\to R^{C}$ near a specific input instance $x\in R^{d}$ with a locally linear interpretable model g∈G , such as a sparse linear regressor. The goal is to minimize the following objective:

$$L\left(f,g,\pi_{x}\right)+\Omega_{\left(g\right)}$$

Here:

• L is a loss function measuring the fidelity of g in approximating f within the local neighbourhood $\pi_{x}$ around x,
• Ω penalizes model complexity to encourage interpretability,
• πx(z) is a locality-aware kernel function weighting the importance of perturbed samples z based on their proximity to x.

In our image domain setting, the original image $x\in R^{H\times W\times 3}$ is segmented into super pixels $\left\{s_{1},s_{2},...,s_{k}\right\}$ . Perturbations $z\in {\left\{0,1\right\}}^{k}$ are generated by randomly turning super pixels “on” or “off,” and the model’s predictions on these perturbed instances are collected to learn the local surrogate model g.

#### 5.9.2 Image perturbation and prediction pipeline

Each image was normalized to [0, 1] and resized to a standard dimension of 128 × 128. The model’s softmax prediction function is defined as:

$$f\left(x\right)=soft\max\left(F\left(x\right)\right)=\left[\frac{e^{F_{1}\left(x\right)}}{\sum_{c=1}^{C}e^{F_{c}\left(x\right)}},...,\frac{e^{F_{c}\left(x\right)}}{\sum_{c=1}^{C}e^{F_{c}\left(x\right)}}\right]$$

Where $f\left(x\right)\in R^{C}$ denotes the logits (unnormalized predictions) for C classes. For each image, LIME generates n = 1000 perturbed samples $\left\{z^{\left(i\right)}\right\}$ and uses the corresponding predictions $\left\{f\left(z^{\left(i\right)}\right)\right\}$ to fit a sparse linear model: $g\left(z\right)=\omega^{T}z+b$ where ${\left\|\omega \right\|}_{0}\leq K$ .

Here, K is the number of features (super pixels) used in the explanation, constrained to = 5 in our experiments for enhanced interpretability.

#### 5.9.3 Visualization and results interpretation

For both ALL Blood Smear and CNMC Classification datasets, LIME was used to generate super pixel-level explanations for the top-1 predicted class across three representative images per class per optimizer (Nadam, SGD, and Fractional). The interpretable regions were visualized by overlaying masks on the input images, highlighting the positive contributing areas with boundary contours.

Key observations include:

• Optimizers with Balanced Attention (Nadam, Fractional): These models consistently attributed their decisions to biologically relevant structures. For instance, in leukemic images, LIME explanations emphasized irregular chromatin patches, enlarged nuclei, and cytoplasmic granularity, features clinically correlated with malignancy.
• CNN-Heavy Models (SGD): LIME highlighted more localized, texture-dominant super pixels, suggesting reduced contextual awareness. While this strategy yielded strong classification performance, it may hinder generalization in noisy or low-contrast scenarios.
• Consistency Across Folds: Across multiple folds, explanations for correctly classified samples remained stable, which reinforces the robustness and trustworthiness of the learned features.

**FIGURE 21:**
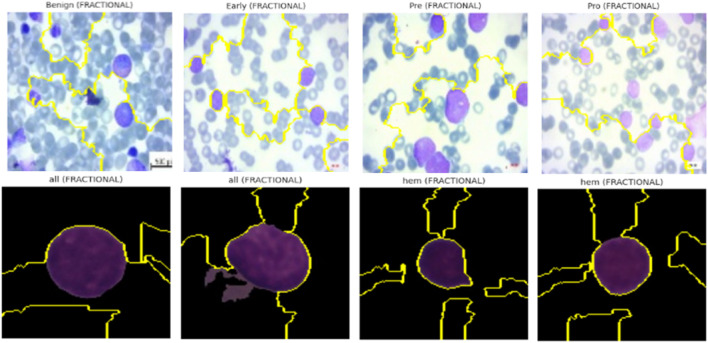
LIME-based XAI visualizations.

The explainability visualization of the proposed model using LIME are shown in Figure 21.

#### 5.9.4 Complementarity with Grad-CAM

Whereas Grad-CAM provides class-specific attention maps derived from internal gradients, LIME supplements this by modelling the influence of pixel groupings directly at the input level using a local surrogate model. This dual-view interpretability, internal (Grad-CAM) and external (LIME), ensures that the model decisions align with both feature hierarchies and raw input signal patterns, increasing clinical confidence in AI-assisted diagnosis.

### 5.10 Integrated Gradients for model interpretability

The Causal-Former-HMC model’s decision-making process was made even more transparent by using Integrated Gradients (IG), an attribution-based interpretability technique. By integrating the gradients of the model’s output along a path from a baseline (such as a black image) to the input image, IG quantifies the contribution of each input feature (in this case, image pixels).

#### 5.10.1 Theoretical formulation of Integrated Gradients

Let $u:R^{d}\to R$ be the predictive model and $z\in R^{d}$ the input vector (flattened RGB image). Let $\tilde{z}\in R^{d}$ denote a baseline input (typically a zero or grayscale image). The integrated gradient of the *w*th feature is defined as:

$$I_{nt}Gr_{w}\left(z\right)=\left(z_{w}-{\tilde{z}}_{w}\right)\times \int_{\nu =0}^{1}\frac{\partial u\left(\tilde{z}+\nu \left(z-\tilde{z}\right)\right)}{\partial u_{w}}d\nu$$

The accumulation of gradients along the straight-line path from baseline $\tilde{z}$ to input z is calculated by this equation. Riemann sums with n stages are used to quantitatively approximate the integral:

$$I_{nt}Gr_{w}\left(z\right)\approx \left(z_{w}-{\tilde{z}}_{w}\right)\times \frac{1}{n}\sum_{k=1}^{n}\frac{\partial u\left(\tilde{z}+\frac{k}{n}\left(z-\tilde{z}\right)\right)}{\partial z_{w}}$$

This method satisfies the axioms of Sensitivity and Implementation Invariance, which are desirable properties for any attribution method.

#### 5.10.2 Attribution workflow

For each image in the ALL and CNMC datasets, the following pipeline was applied:

• Each image $z\in R^{H\times W\times 3}$ was resized to 128 × 128 and normalized to [0, 1].
• A baseline input $\tilde{z}$ was selected as a black image of the same shape.
• The IG method was applied using n = 50 steps to approximate the integral.
• The resulting attribution map IG(z) was normalized to the range [0, 1] for visual representation.

Mathematically, the tensor-based approximation used in the experiments is computed via:

$$I_{nt}Gr\left(z\right)=\left(z-\tilde{z}\right)⊙\frac{1}{n}\sum_{k=1}^{n}\nabla_{z}u\left(\tilde{z}+\frac{k}{n}\left(z-\tilde{z}\right)\right)$$

where ⊙ denotes element-wise multiplication.

#### 5.10.3 Interpretability and visual evidence

Visualizations were generated for three representative images from each class. For each image:

• The left pane displayed the original image.
• The right pane showed the IG attribution map, where pixel intensity corresponded to the importance of that region in the final prediction.
Key interpretative insights include:
• For benign samples, attributions were predominantly uniform, focusing on non-leukocytic regions.
• For malignant subtypes (Early, Pre, Pro), the model heavily emphasized morphological features such as nuclear contour irregularity and chromatin distribution.
• Attribution patterns remained consistent across optimizers like Nadam, SGD, and Fractional, but the relative sharpness and localization of attributions varied, reflecting optimizer-induced variance in representational focus.

**FIGURE 22:**
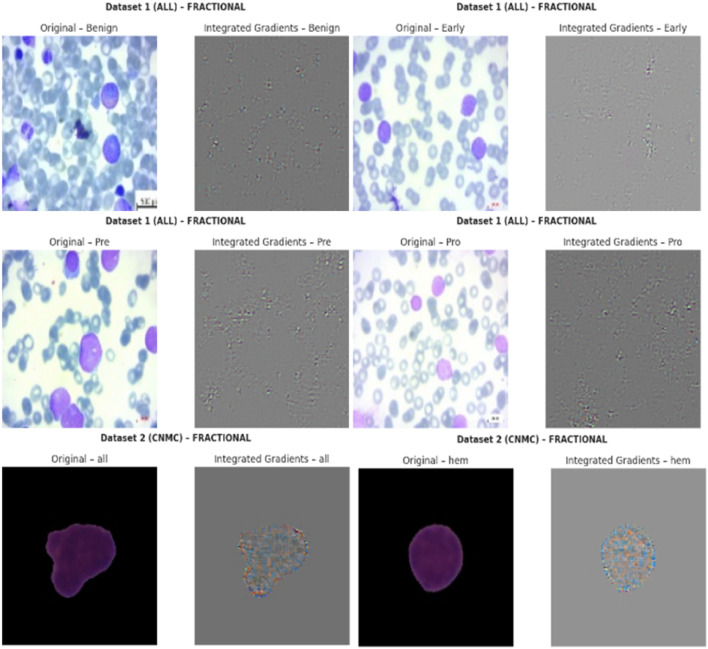
Integrated gradients-based XAI visualizations.

The IG-based XAI visualization of the proposed model’s predications are shown in Figure 22.

#### 5.10.4 Clinical and diagnostic implications

The IG maps revealed that the model attends to biologically meaningful regions, thereby enhancing its explainability in diagnostic contexts. Such transparency is particularly crucial in medical AI, where decisions must be both accurate and accountable. By integrating over a path from neutral baselines, IG provides a complete and axiomatic attribution that helps validate the model’s trustworthiness to clinicians and stakeholders.

### 5.11 SHAP overlay visualizations for model interpretability

To further assess the interpretability of the proposed Causal-Former-HMC architecture, SHAP (SHapley Additive exPlanations) was applied to generate class-discriminative overlay heatmaps. SHAP offers a theoretically grounded framework derived from cooperative game theory, dividing a model’s output by the marginal contribution of each input feature. When applied to deep learning models, SHAP helps in identifying salient regions within medical images that significantly influence the model’s predictions.

#### 5.11.1 Mathematical framework of SHAP

SHAP explanations are built upon the Shapley value $\phi_{j}$ , which illustrates the feature’s contribution j across all possible feature subsets $S\subseteq E\backslash \left\{j\right\}$ , where E represents the set of all input features. The formal definition is:

$$\phi_{j}\left(f,z\right)=\sum_{S\subseteq E\backslash \left\{j\right\}}\frac{\left|S\right|!\left(\left|E\right|-\left|S\right|-1\right)!}{\left|E\right|!}\left[f\left(z_{S\cup \left\{j\right\}}\right)-f\left(z_{s}\right)\right]$$

Here, $f\left(z_{S}\right)$ represents the model output when only the features in subset S are observed (others replaced with a baseline). This expression ensures fairness, as it considers all possible coalitions of features.

#### 5.11.2 SHAP with gradient explainer in deep models

For neural networks, SHAP approximates the Shapley values via gradient-based methods. The Gradient SHAP explainer used in this study estimates the contribution of input features by computing the expected gradients with respect to background samples.

Let $f:R^{H\times W\times C}\to R^{K}$ be a model outputting class logit, and let x be the input image. The SHAP value for pixel i is approximated as:

$$\phi_{i}\left(x\right)\approx E_{\tilde{x}∼B}\left[\left(x_{i}-{\tilde{x}}_{i}\right).\int_{\alpha =0}^{1}\frac{\partial f\left(\tilde{x}+\alpha \left(x-\tilde{x}\right)\right)d\alpha }{\partial x_{i}}\right]$$

Where $\tilde{x}\in B$ is sampled from a background distribution B, which in this study consisted of randomly selected images from the respective dataset. This ensures robustness by mitigating attribution noise through averaging.

#### 5.11.3 Visualization pipeline

For each class in the ALL and CNMC datasets, the following steps were undertaken:

• A batch of three class-specific images was selected.
• Each image x was transformed into a tensor and passed through the model to compute class-wise SHAP values.
• The SHAP attribution maps were averaged across colour channels and normalized to a [0, 1] range.
• The normalized maps were converted into RGB heatmaps using a jet colormap, and then overlaid on the original images for visual interpretation.

The final overlay map M was generated using a weighted sum:

$$M=\lambda .x+\left(1-\lambda \right).H$$

Where:

• x is the original normalized image,
• H is the SHAP heatmap,
• λ = 0.6 is the blending coefficient used to retain visual clarity.

**FIGURE 23:**
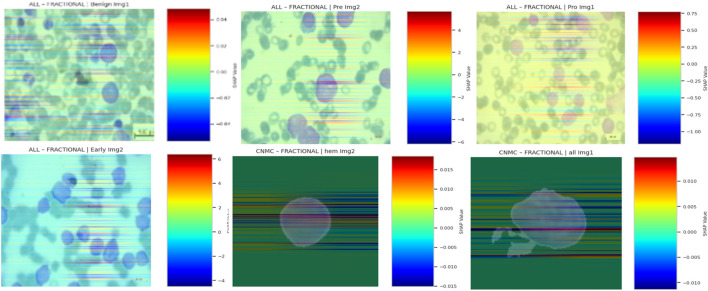
SHAP-based XAI visualizations.

Finally, the SHAP-based explainable AI visualizations of the proposed model’s predictions are shown in Figure 23.

#### 5.11.4 Interpretive insights

SHAP overlays across optimizers and datasets consistently highlighted domain-relevant features:
• In benign cells, SHAP emphasized cytoplasmic boundaries and uniform textures.
• In leukemic subtypes, focal regions included nuclear membrane disruptions, chromatin condensation, and atypical nucleoli, features indicative of malignancy in haematological microscopy.
• Across optimizers, SGD-trained models exhibited more sharply focused attributions, while Nadam and Fractional maintained broader but still relevant activation distributions.

These overlays confirmed that the model internalized clinically meaningful patterns, and not superficial correlations or imaging artifacts. By tracing predictions to interpretable pixel regions, SHAP overlays strengthen the diagnostic credibility of the proposed hybrid causal architecture.

To evaluate interpretability, we applied four *post hoc* explainable AI techniques: Grad-CAM, LIME, Integrated Gradients (IG), and SHAP. Among these, Grad-CAM consistently highlighted clinically relevant nuclear and chromatin regions in an intuitive heatmap form that was readily interpretable by pathologists. SHAP further strengthened interpretability by providing pixel-level attributions with a solid theoretical foundation in cooperative game theory, ensuring consistency across patient samples. While LIME and IG provided useful local explanations, they were occasionally less stable across folds and more sensitive to perturbation noise. Overall, the complementary use of Grad-CAM (visual clarity) and SHAP (theoretical rigor) offered the most clinically trusted and reproducible interpretability, thereby supporting the deployment of the proposed framework in hematology workflows.

## 6 Conclusion

This study introduced Causal-Former-HMC, a groundbreaking hybrid AI model that brings together convolutional neural networks (CNNs), vision transformers (ViTs), and a causal graph learner with counterfactual reasoning to tackle the challenge of diagnosing Acute Lymphoblastic Leukemia (ALL) using peripheral blood smear (PBS) images. The results speak for themselves: the model delivered perfect classification accuracy (100%) and macro-averaged F1-scores on the ALL Image dataset, while achieving up to 98.5% accuracy with a stellar 0.9975 ROC-AUC on the C-NMC dataset. These results, confirmed with stratified 5-fold cross-validation and multiple optimizers (Nadam, SGD, and RAdam), demonstrate the model’s stability and capacity to generalize across diverse datasets. Beyond raw performance, interpretability was a key component of this effort. We ensured that the model’s judgments were comprehensible by utilizing advanced explainable AI (XAI) approaches such as Grad-CAM, LIME, Integrated Gradients, and SHAP, while also highlighting clinically significant aspects such as nuclear contour abnormalities and chromatin condensation.

The relevance of these findings goes far beyond the numbers. Causal-Former-HMC, with its high accuracy and straightforward decision-making process, is a potential technique for non-invasive leukemia screening. Traditional diagnostics, such as bone marrow biopsies, are invasive, expensive, and rely on expert interpretation, which this approach avoids by utilizing widely available PBS pictures. This could result in speedier, more accessible diagnoses, especially in resource-limited settings, as well as a move toward earlier identification and improved patient outcomes. The model’s capacity to discern small changes between leukemic subtypes suggests that it could promote individualized treatment options, which are crucial in clinical hematology.

Looking ahead, there is lots of room to expand on this basis. One important step would be to run the model on larger, more diverse datasets. While the ALL and C-NMC datasets were a good starting point, adding photos from different populations and healthcare contexts would prove the model’s reliability in real-world circumstances. Another fascinating area is to integrate multimodal data, such as PBS images with health records or genomic profiles. This could improve diagnostic precision even further, providing a more complete picture of the disease and possibly even predicting treatment outcomes.

Real-world deployment is another priority. Testing Causal-Former-HMC in live clinical situations would reveal how well it integrates into existing processes, how fast it is in practice, and how clinicians react to its results. Pilot trials in diagnostic labs could improve its usability and overcome any practical challenges. Beyond ALL, the model’s architecture might be adapted to other leukemias or blood diseases, increasing its reach. The combination of local and global feature extraction with causal reasoning appears adaptable enough to address comparable hematology challenges.

There is also room to improve the interpretability angle. While current XAI techniques are effective, creating new methods suited to medical imaging could delve deeper into the model’s thinking, potentially revealing new diagnostic insights or biomarkers. This could make the tool much more important to clinicians who must believe and act on its findings.

While the proposed Causal-Former-HMC model demonstrates promising results, several limitations must be acknowledged. First, the datasets employed (ALL and C-NMC) are relatively modest in size and originate from limited sources, which may restrict the generalizability of the findings. The reported 100% accuracy on the ALL dataset, although encouraging, raises concerns about potential overfitting, particularly in homogeneous data settings. Furthermore, both datasets represent controlled environments that may not fully capture the variability present in multi-center clinical workflows, such as differences in staining protocols, imaging devices, or patient demographics. To strengthen the robustness and translational potential of the model, future work should include large-scale, multi-center validation across diverse populations and real-world laboratory conditions.

Despite the strong experimental results, several practical considerations must be acknowledged before clinical deployment. Real-world peripheral blood smear (PBS) images often exhibit variability in staining quality, imaging devices, and preparation protocols, which may affect model reliability. Integration into workflows will require a clinician-in-the-loop approach to ensure that predictions complement rather than replace expert judgment. Furthermore, the use of AI in clinical diagnostics is subject to regulatory and ethical approvals, which can be complex and time-consuming. Finally, while our model was optimized for efficiency, the computational cost of transformer-based architectures may still pose challenges in low-resource healthcare settings. Addressing these factors through multi-center validation, workflow integration studies, and computational optimization will be critical to achieving real-world clinical impact.

In conclusion, this work represents a significant step forward in AI-driven leukemia detection. Causal-Former-HMC combines superior accuracy with the transparency that clinicians require, paving the way for its application in real-world care. Future efforts should focus on scaling it up, testing it in practice, and realizing its full promise in hematology, bringing us closer to smarter, more accessible diagnostics.

## Data Availability

Publicly available datasets were analyzed in this study. This data can be found here: The datasets analyzed during this study are publicly available on Kaggle. Dataset 1, titled “Leukemia” by Mehrad Aria, can be accessed at https://www.kaggle.com/datasets/mehradaria/leukemia. Dataset 2, titled “Leukemia Classification” by Andrew MVD, is available at https://www.kaggle.com/datasets/andrewmvd/leukemia-classification. These resources were utilized to support the findings of this research.

## References

[B23] Aby A. E. Salaji S. Anilkumar K. K. Rajan T. (2022). A review on leukemia detection and classification using Artificial Intelligence-based techniques. Comput. Biol. Med. 147, 105762. 10.1016/j.compeleceng.2024.109446

[B1] Ahmed S. Shoukat S. Shehzad K. Ahmad I. Eshmawi A. A. Amin A. H. (2022). A deep learning-based approach for the diagnosis of acute lymphoblastic leukemia. Electronics 11 (19), 3168. 10.3390/electronics11193168

[B24] Alcazer V. Le Meur G. Roccon M. Barriere S. Le Calvez B. Badaoui B. (2024). Evaluation of a machine-learning model based on laboratory parameters for the prediction of acute leukaemia subtypes: a multicentre model development. Lancet Digital Health 6 (4), e234–e245. 10.1186/s12885-024-12646-3 38670741

[B13] Al-Obeidat F. Hafez W. Rashid A. Jallo M. K. Gador M. Cherrez-Ojeda I. (2025). Artificial intelligence for the detection of acute myeloid leukemia from microscopic blood images; a systematic review and meta-analysis. Front. Big Data 7, 1402926. 10.3389/fdata.2024.1402926 39897067 PMC11782132

[B28] Ansari S. Navin A. H. Sangar A. B. Gharamaleki J. V. Danishvar S. (2023). A customized efficient deep learning model for the diagnosis of acute leukemia cells based on lymphocyte and monocyte images. Sensors 23 (2), 322. 10.3390/applbiosci4010009

[B3] Aria M. Ghaderzadeh M. Bashash D. Abolghasemi H. Asadi F. Hosseini A. (2021). Acute lymphoblastic leukemia (ALL) image dataset. 10.34740/kaggle/dsv/2175623

[B4] Bain B. J. Leach M. (2024). Artificial intelligence-based management of adult chronic myeloid leukemia: where are We and where are We going? Front. Hematol. 6, 10930728. 10.3390/diagnostics13071330 PMC10930728 38473210

[B5] Bain B. J. Momeni-Boroujeni A. Rios-Doria E. Abu-Rustum N. Soslow R. A. (2023). The significance of International Federation of Gynecology and Obstetrics grading in Microsatellite instability-high and POLE-Mutant Endometrioid Endometrial Carcinoma. Mod. Pathol. 36 (10), 100234. 10.1016/j.modpat.2023.100234 37268062 PMC10528952

[B6] Bhojwani D. Yang J. J. Pui C.-H. (2015). Biology of childhood acute lymphoblastic leukemia. Pediatr. Clin. N. Am. 62 (1), 47–60. 10.1016/j.pcl.2014.09.004 25435111 PMC4250840

[B7] Chaurasia V. Teng P. Vyapari K. Banola A. Foster G. Diaz E. (2024). Quantifying lung fissure integrity using a three-dimensional patch-based convolutional neural network on CT images for emphysema treatment planning. J. Med. Imaging 11 (3), 034502. 10.1117/1.JMI.11.3.034502 PMC11135203 38817711

[B8] Chen Y.-M. Chou F.-I. Ho W.-H. Tsai J.-T. (2025). Deep learning-based detection and classification of acute lymphoblastic leukemia with explainable AI techniques. Array 25, 100397. 10.1016/j.array.2025.100397

[B26] Cheng F.-M. Lo S.-C. Lin C.-C. Lo W.-J. Chien S.-Y. Sun T.-H. (2024). Deep learning assists in acute leukemia detection and cell classification via flow cytometry using the acute leukemia orientation tube. Sci. Rep. 14 (1), 58580. 10.1038/s41598-024-59259-1 PMC11004172 38594383

[B2] Didi I. Alliot J. M. Dumas P. Y. Vergez F. Tavitian S. Largeaud L. (2024). Artificial intelligence-based prediction models for acute myeloid leukemia using real-life data: a DATAML registry study. Leukemia Res. 136, 107437. 10.1016/j.leukres.2024.107437 38215555

[B10] Dutta M. Mojumdar M. U. Kabir M. A. Chakraborty N. R. Siddiquee S. M. T. Abdullah S. (2023). “LEU3: an attention Augmented-based model for acute lymphoblastic leukemia classification,” in Proc. IEEE. 10.1109/ACCESS.2025.3542609

[B11] Elsayed B. Elshoeibi A. Elhadary M. Badr A. Metwalli O. Cherif H. (2022). Deep learning models for the diagnosis of acute lymphoblastic leukemia from bone marrow images: a comprehensive literature review. Blood 140 (Suppl. 1), 7184. 10.1182/blood-2023-187037

[B12] Elsayed B. Elhadary M. Elshoeibi A. Badr A. Metwally O. (2023). Deep learning enhances acute lymphoblastic leukemia diagnosis and classification using bone marrow images. Front. Oncol. 13, 1330977. 10.3389/fonc.2023.1330977 38125946 PMC10731043

[B15] Gökbuget N. Boissel N. Chiaretti S. Dombret H. Doubek M. Fielding A. (2024). Diagnosis, prognostic factors, and assessment of ALL in adults: 2024 ELN recommendations from a European expert panel. Blood 143 (19), 1891–1902. 10.1182/blood.2023020794 38295337

[B16] Gupta A. Gupta R. (2019). ALL Challenge dataset of ISBI 2019. Cancer Imaging Archive. 10.7937/tcia.2019.dc64i46r

[B17] Gupta A. Duggal R. Gehlot S. Gupta R. Mangal A. Kumar L. (2020). GCTI-SN: Geometry-inspired chemical and tissue invariant stain normalization of microscopic medical images. Med. Image Anal. 65, 101788. 10.1016/j.media.2020.101788 32745978

[B9] Gupta R. Gehlot S. Gupta A. (2022). C-NMC: B-lineage acute lymphoblastic leukemia: a blood cancer dataset. Comput. Methods Programs Biomed. 222, 106943. 10.1016/j.medengphy.2022.103793 35500994

[B18] Gupta A. Gupta R. (2024). ALL-Net: integrating CNN and explainable-AI for enhanced diagnosis and interpretation of acute lymphoblastic leukemia. PeerJ Comput. Sci. 10, e11888852. 10.7717/peerj-cs.1852 PMC11888852 40062280

[B19] Huang M.-L. Huang Z.-B. (2024). An attention-based deep learning for acute lymphoblastic leukemia classification. Sci. Rep. 14 (1), 67826. 10.1038/s41598-024-58452-6 PMC11286757 39075091

[B20] Jammal F. Dahab M. Bayahya A. Y. (2025). Neuro-bridge-X: a neuro-Symbolic vision transformer with meta-XAI for interpretable leukemia diagnosis from peripheral blood smears. Diagnostics 15 (16), 2040. 10.3390/diagnostics15162040 40870894 PMC12385239

[B21] Jiwani N. Gupta K. Pau G. Alibakhshikenari M. (2023). Pattern recognition of acute lymphoblastic leukemia (ALL) using computational deep learning. IEEE Access 11, 29541–29553. 10.1109/access.2023.3260065

[B22] Jiwani N. Gupta K. Pau G. Alibakhshikenari M. (2024). Deep learning algorithms for early diagnosis of acute lymphoblastic leukemia. arXiv Prepr. arXiv:2407.10251. 10.1109/ACCESS.2023.3260065

[B27] Rahman R. Sun X. Huang T. Song K. (2022). A new self-calibration and Compensation method for Installation Errors of Uniaxial rotation module Inertial Navigation system. Sensors 22 (10), 3812. 10.3390/s22103812 35632221 PMC9143706

[B14] Talaat F. M. Gamel S.A. (2023). Machine learning in detection and classification of leukemia using C-NMC_Leukemia. Multimedia Tools Appl. 82 (23), 34567–34589. 10.1007/s11042-023-15683-5

[B25] Wang Y. Lam H. K. Hou Z. G. Li R. Q. Xie X. L. Liu S. Q. (2023). Deep learning for leukemia classification using microscopic blood images. Med. Image Anal. 89, 102876. 10.1016/j.media.2023.102876 37423057

[B29] Ward E. DeSantis C. Robbins A. Kohler B. Jemal A. (2014). Childhood and adolescent cancer statistics. CA A Cancer J. Clin. 64 (2), 83–103. 10.3322/caac.21219 24488779

[B30] Wu Y. Gadsden S. A. (2022). A review of artificial intelligence applications in hematology Management: current practices and future Prospects. Front. Artif. Intell. 5, 987654. 10.2196/36490 PMC9328784 35819826

[B31] Wu Y. Gadsden S. A. (2024). Artificial intelligence reveals the predictions of hematological indexes in children with acute leukemia. BMC Cancer 24 (1), 12646. 10.1186/s12885-024-12256-z PMC11318239 39134989

